# Finerenone Attenuates Endothelial Dysfunction and Albuminuria in a Chronic Kidney Disease Model by a Reduction in Oxidative Stress

**DOI:** 10.3389/fphar.2018.01131

**Published:** 2018-10-09

**Authors:** Raquel González-Blázquez, Beatriz Somoza, Marta Gil-Ortega, Miriam Martín Ramos, David Ramiro-Cortijo, Elena Vega-Martín, Angela Schulz, Luis Miguel Ruilope, Peter Kolkhof, Reinhold Kreutz, María S. Fernández-Alfonso

**Affiliations:** ^1^Departamento de Ciencias Farmacéuticas y de la Salud, Facultad de Farmacia, Universidad San Pablo-CEU, Madrid, Spain; ^2^Instituto Pluridisciplinar and Facultad de Farmacia, Universidad Complutense de Madrid, Madrid, Spain; ^3^Department of Physiology, Universidad Autónoma de Madrid, Madrid, Spain; ^4^Department of Clinical Pharmacology and Toxicology, Berlin Institute of Health, Charité – Universitätsmedizin Berlin, Corporate Member of Freie Universität Berlin, Humboldt-Universität zu Berlin, Berlin, Germany; ^5^Unidad de Hipertensión, Instituto de Investigación Imas12, Hospital Universitario 12 de Octubre, Madrid, Spain; ^6^Departamento de Medicina Preventiva y Salud Pública, Universidad Autónoma de Madrid, Madrid, Spain; ^7^Drug Discovery, Pharmaceuticals, Cardiology Research, Bayer HealthCare AG, Wuppertal, Germany

**Keywords:** albuminuria, finerenone, aldosterone antagonists, aorta endothelial dysfunction, oxidative stress

## Abstract

Albuminuria is an early marker of renovascular damage associated to an increase in oxidative stress. The Munich Wistar Frömter (MWF) rat is a model of chronic kidney disease (CKD), which exhibits endothelial dysfunction associated to low nitric oxide availability. We hypothesize that the new highly selective, non-steroidal mineralocorticoid receptor (MR) antagonist, finerenone, reverses both endothelial dysfunction and microalbuminuria. Twelve-week-old MWF (MWF-C; MWF-FIN) and aged-matched normoalbuminuric Wistar (W-C; W-FIN) rats were treated with finerenone (FIN, 10 mg/kg/day p.o.) or vehicle (C) for 4-week. Systolic blood pressure (SBP) and albuminuria were determined the last day of treatment. Finerenone lowered albuminuria by >40% and significantly reduced SBP in MWF. Aortic rings of MWF-C showed higher contractions to either noradrenaline (NA) or angiotensin II (Ang II), and lower relaxation to acetylcholine (Ach) than W-C rings. These alterations were reversed by finerenone to W-C control levels due to an upregulation in phosphorylated Akt and eNOS, and an increase in NO availability. Apocynin and 3-amino-1,2,4-triazole significantly reduced contractions to NA or Ang II in MWF-C, but not in MWF-FIN rings. Accordingly, a significant increase of Mn-superoxide dismutase (SOD) and Cu/Zn-SOD protein levels were observed in rings of MWF-FIN, without differences in p22phox, p47phox or catalase levels. Total SOD activity was increased in kidneys from MWF-FIN rats. In conclusion, finerenone improves endothelial dysfunction through an enhancement in NO bioavailability and a decrease in superoxide anion levels due to an upregulation in SOD activity. This is associated with an increase in renal SOD activity and a reduction of albuminuria.

## Introduction

Albuminuria is a hallmark of both early kidney damage and progression of chronic kidney disease (CKD) in non-diabetic and diabetic subjects (Eckardt et al., 2013; Carrero et al., 2017). In addition, albuminuria is an independent risk factor for cardiovascular and all-cause mortality in the general population (Gansevoort et al., 2010; Matsushita et al., 2010). In patients with diabetes mellitus and hypertension, increased urinary albumin excretion (UAE) in a range even below the lower clinical cut of defining albuminuria (i.e., 30 mg/24 h) predicts cardiovascular events and a continuous relationship between cardiovascular and non-cardiovascular mortality has been reported (Piepoli et al., 2016). Thus, UAE does not only reflect a prognostic marker in these patients but might also represent an important target for treatment decisions including blood pressure control with antihypertensive treatment (Pascual et al., 2014; Piepoli et al., 2016). Patients with albuminuria exhibit a widespread vascular damage (Fogarty et al., 2000; Freedman et al., 2008) due to a systemic endothelial disorder also affecting the glomerular endothelium (Deckert et al., 1989; Stehouwer and Smulders, 2006). Endothelial dysfunction has been linked to an increased glomerular and vascular oxidative stress in patients with albuminuria (Vaziri et al., 2002; Satoh, 2012). Data from our group show that patients with resistant albuminuria, developed under adequate chronic pharmacologic blockade of the renin–angiotensin system, exhibit an enhanced oxidative stress that is not well counterbalanced by an adequate endogenous antioxidant defense (Ruiz-Hurtado et al., 2014). Endothelial dysfunction associated to oxidative stress may thus represent a common pathophysiological mechanism leading to both cardiovascular and renal disease.

The Munich Wistar Frömter (MWF) rat is an experimental genetic model of spontaneous non-diabetic albuminuria development and renal injury that mirrors several features observed in patients with albuminuria and CKD (van Es et al., 2011; Schulz and Kreutz, 2012). In MWF, the reduction of the nephron number is inherited and compensatory glomerular hypertrophy is already present at 4 weeks of age due to hyperfiltration, whereas albuminuria is detected at 6 weeks of age (Ijpelaar et al., 2008) preceding the development of hypertension and endothelial dysfunction (Gschwend et al., 2002a; Ulu et al., 2009; Szymanski et al., 2012; Steireif et al., 2013; Gil-Ortega et al., 2015). The latter is observed in the aorta (Ulu et al., 2009; Szymanski et al., 2012; Steireif et al., 2013), mesenteric arteries (Gschwend et al., 2002b; Gil-Ortega et al., 2015) and coronary microvessels (Gschwend et al., 2002b) at 12 weeks of age and was associated with a low nitric oxide (NO) bioavailability associated to an enhanced superoxide anion (O_2_^-^) production (Steireif et al., 2013).

Aldosterone contributes to the development of endothelial dysfunction and the progression of cardiovascular and renal disease (Hirono et al., 2007; Thum et al., 2011). It increases oxidative stress in the vascular wall in a mineralocorticoid receptor (MR)-dependent manner (Briet and Schiffrin, 2013). Currently, available steroidal MR antagonists (MRAs), i.e., spironolactone and eplerenone, have been proven clinically effective in patients with CKD (Bolignano et al., 2014) and demonstrated beneficial effects in different models of hypertension by ameliorating endothelial dysfunction (Quaschning et al., 2001; Sanz-Rosa et al., 2005) and reducing oxidative stress (Quaschning et al., 2001; Virdis et al., 2002; Sanz-Rosa et al., 2005). However, the use of steroidal MRAs is limited and often discontinued in patients with severe CKD, due to their potential to the increase the risk of severe hyperkalemia and worsening renal function (Khosla et al., 2009).

Finerenone (BAY 94-8862) is a novel non-steroidal MRA, with a similar potency to spironolactone and even more selective than eplerenone, at least 500-fold toward MR (Barfacker et al., 2012). The non-steroidal structure of finerenone has a strong impact on its binding mode within MR (Amazit et al., 2015), as well as physicochemical properties like lipophilicity and polarity, which determine plasma protein binding, transport, tissue penetration and distribution (Kolkhof et al., 2015). Quantitative whole-body autoradiography showed a balanced distribution of finerenone into rat heart and kidney, which contrasts with the distribution pattern of steroidal MRAs in rodents (Kolkhof et al., 2014). Treatment with finerenone prevents functional and structural damage in heart and kidney from deoxycorticosterone acetate (DOCA)/salt challenged rats at non-blood pressure lowering doses (Kolkhof et al., 2014). In patients with chronic heart failure and concomitant mild-to-moderate CKD, finerenone reduced albuminuria to the same magnitude as spironolactone, with a significantly smaller mean increase in serum potassium concentration and a smaller decrease in eGFR (Pitt et al., 2013).

The hypothesis of this study is that finerenone treatment both reverses endothelial dysfunction and albuminuria through a reduction of oxidative stress in the vascular wall. For this purpose, we have analyzed the effect of a 4-week treatment with finerenone (10 mg/kg/day) on the relationship between increased UAE, oxidative stress and vascular dysfunction in 16 weeks-old MWF and their aged-matched normoalbuminuric controls from the Wistar (W) strain.

## Materials and Methods

### Animals and Experimental Protocol

Twelve-week-old male Wistar (W; Charles River, Barcelona, Spain) and MWF rats (Charité – University Medicine Berlin, Germany) were housed in groups of two under controlled dark-light cycles (12 h/12 h), temperature conditions and with food (A.04, Panlab) and water available *ad libitum*. Animals were randomly grouped to receive finerenone (10 mg/kg/day in 10% EtOH, 40% PEG400, 50% water; W-FIN; MWF-FIN; *n* = 10 per group) or vehicle (10% EtOH, 40% PEG400, 50% water; W-C; MWF-C; *n* = 10 per group) during 4 weeks by once daily oral gavage. Last oral administration of FIN was performed the day before sacrifice.

Urinary albumin excretion was determined placing the animals in metabolic cages for 24 h after a 1-day adaptation period. UAE was measured by enzyme-linked immunosorbent assay (ELISA) using a rat specific antibody (ICN Biomedicals, Eschwege, Germany). The day before sacrifice, tail vein blood was obtained 90–120 min after oral administration of finerenone to assess drug plasma concentrations by HPLC-MS. Blood was collected in heparin lithium tubes and centrifuged at 900 *g* for 10 min at 4°C to obtain plasma that was frozen at -80°C. Aldosterone levels were determined by RAAS Triple-A Analysis (Attoquant Diagnostics GmbH, Vienna, Austria).

Blood pressure (BP) was measured at the end of treatment by the tail-cuff method after a previous adaptation to the cuff (twice a week for 3 weeks). Direct BP determination was performed in rats anesthetized with ketamine (Imalgene 1000, Merial; 80 mg/kg i.p.) and xylazine (Rompun 2%, Bayer; 8 mg/kg i.p.) after cannulation of the carotid artery using a 0.58/0.97 mm (inner- and outer-diameter) catheter. The catheter was connected to a data acquisition system (PowerLab 4/30, ADInstruments, United Kingdom) and signals were digitally stored for analysis using the LabChart 7.0 Pro software. Mean arterial blood pressure was analyzed. After hemodynamic measurements, animals were sacrificed and tissues were removed for study. All experimental procedures were approved by the Institutional Animal Care and Use Committee according to the guidelines for ethical care of experimental animals of the European Community.

### Vascular Reactivity in the Isolated Thoracic Aorta

Thoracic aorta was carefully isolated, placed in oxygenated physiological salt solution (PSS), and cleaned of blood and perivascular fat. Vascular rings (3-mm-long) were suspended on two intraluminal parallel wires, introduced in an organ bath containing PSS (115 mmol/L NaCl, 4.6 mmol/L KCl, 2.5 mmol/L CaCl_2_, 25 mmol/L NaHCO_3_, 1.2 mmol/L KH_2_PO_4_, 1.2 mmol/L MgSO_4_, 0.01 mmol/L EDTA, 5.5 mmol/L glucose) and connected to a Piodem strain gauge. Isometric tension was recorded in a Power Lab system (ADInstruments, Oxford, United Kingdom). Segments were given an optimal resting tension of 1.5 g, which is then readjusted every 15 min during a 90-min equilibration period. Thereafter, the vessels were exposed to 75 mmol/L KCl to check their contractility. Contraction curves to noradrenaline (NA, 10^-10^–5 × 10^-7^ mol/L) and angiotensin II (Ang II 10^-9^–5 × 10^-7^ mol/L) were performed. The nitric oxide synthase inhibitor, N_G_-nitro-L-arginine methyl ester (L-NAME, 10^-4^ mmol/L), the NOX inhibitor, apocynin (10^-4^ mmol/L) or the catalase inhibitor, 3-amino-1,2,4-triazole (3-AT, 5 × 10^-3^ mmol/L) were incubated 30 min prior to addition of the agonists. Relaxation curves to acetylcholine (Ach, 10^-9^–10^-4^mol/L) and sodium nitroprusside (SNP, 10^-12^–10^-5^ mol/L) were performed in segments pre-contracted with NA at concentrations which varied between 10^-7^ and 10^-6^ M to ensure a similar pre-contraction level between groups and treatments. Pre-contraction, expressed as percentage of 75 mM KCl, was: W-C = 107.0 ± 7.3; W-FI*N* = 105.5 ± 7.9; MWF-C = 103.1 ± 3.6; MWF-FI*N* = 103.9 ± 7.5; n.s.

### Western Blot

Amounts of NADPH subunits, p22^phox^ and p47^phox^, catalase (CAT), superoxide dismutase (SOD) isoforms SODs (Cu/Zn-SOD, Mn-SOD, and Ec-SOD), eNOS, peNOS(Ser^1177^), Akt, and pAkt (Ser^473^) were quantified in vascular segments. 30 μg protein samples are separated by 10–15% SDS-PAGE gels as appropriate. Primary antibodies against p22^phox^ (1:200 final dilution; Santa Cruz Biotechnology, Germany), p47^phox^ (1:200 final dilution; Santa Cruz Biotechnology, Germany), CAT (1:2000 final dilution; Sigma-Aldrich, Spain), Cu/Zn-SOD (1:100 final dilution; Santa Cruz Biotechnology, Germany), Mn-SOD (1:200 final dilution; Santa Cruz Biotechnology, Germany), ec-SOD (1:1000 final dilution; StressGen, United States), eNOS (1:250 final dilution; Cell Signaling Technology, United States), peNOS^Ser1177^ (1:500 final dilution; Cell Signaling Technology, United States), AKt (1:1000 final dilution; Cell Signaling Technology, United States), and pAKt^Ser473^ (1:1000 final dilution; Cell Signaling Technology, United States) were incubated overnight at 4°C. After washing, appropriate secondary antibodies (anti-rabbit or anti-mouse IgG-peroxidase conjugated) were applied for 1 h. Blots were washed, incubated in commercial enhanced chemiluminescence reagents (ECL, BIO-RAD, Spain) and developed and quantified by using the software Imaging System (Molecular imager^®^ ChemiDoc^TM^ XRS plus, BIO-RAD, Spain). To prove equal loadings of samples, blots were re-incubated with β-actin antibody (Sigma-Aldrich, Spain). Expression values of NADPH subunits, CAT and SOD isoforms were normalized with β-actin to account for variations in gel loading. Expression values of peNOS and pAkt were normalized with eNOS or Akt values, respectively.

### Measurement of Pro-oxidant and Antioxidant Markers

Measurement of pro-oxidant and antioxidant markers in kidney and plasma was quantified as previously reported [9]. Plasma protein carbonyls were measured by the 2,4-dinitrophenylhydrazine-based assay (Hawkins et al., 2009) adapted by our group (Ruiz-Hurtado et al., 2014). The protein carbonyl concentration was assessed using an extinction coefficient of 2,4-dinitrophenylhydrazine (𝜀 = 22,000 M/cm) and expressed as nmol/mg of protein. Protein content was determined by a Coomassie-blue-based microtiter plate assay, according to manufacturer’s instructions (Bio-Rad), and absorbance was measured at 370 nm in a microplate reader (Synergy HTMultimode; BioTek).

Plasma GSH was determined by a fluorimetric assay based on the reaction with o-phthalaldehyde (Hissin and Hilf, 1976) adapted to a microplate reader (Ruiz-Hurtado et al., 2014). Fluorescence was assessed at 360 ± 40 nm excitation and 460 ± 40 nm emission wavelengths and GSH concentration was expressed as μmol/mg of protein. Plasma thiols were determined by a modification of the method based on 5,5^′^-dithiobis(2-nitrobenzoic acid) assay (Hawkins et al., 2009), adapted to a microplate reader (Ruiz-Hurtado et al., 2014). Absorbance was assessed at 412 nm and thiol content was expressed as nM GSH/mg of protein.

Renal total SOD activity was assessed by a modified nitroblue tetrazolium (NBT)-based spectrometric assay including bathocuproine sulfonate (BCS) as chelator agent and inhibitor of mitochondrial electron transport chain, and xanthine oxidase (Spitz and Oberley, 1989). 126 μl BCS/NBT mixture (1 ml BCS 0.13 mM + 34.5 μl NBT 2.6 mM) were mixed with 20 μl homogenate, 20 μl buttermilk xanthine oxidase (0.126 U/ml) and 20 μl potassium phosphate buffer (50 mM, pH 7.5). The mixture was incubated at 37°C and 14 μl xanthine (2.1 mM) were added. The reaction of formazan formation was monitored during 20 min at 630 nm using a microplate reader programmed in kinetic mode. The absorbance change rate (S) was calculated by a linear regression analysis as the absorbance slope vs. the reaction time. SOD activity was expressed in U/mg protein, estimated from the SOD standard curve (0–6 U/ml) obtained as the S_0_/S ratio vs. the enzyme concentration, being S_0_ the slope of the uninhibited (without SOD) reaction. Quantification of plasma superoxide (O_2_^-^) scavenging activity was determined by the SOSA assay adapted to microplate reader. This assay evaluates all the plasma antioxidants capable of eliminating O_2_^-^ and is considered as a global measure of SOD activity [9]. It is based on the inhibition of luminescence emitted by coelenterazine (CTZ) when oxidized by O_2_^-^ [9]. SOSA values were quantified by comparing the luminescence inhibition of each sample with a SOD activity standard curve (0–4 U/ml) and expressed as mU SOD/mg of protein. Catalase activity was determined by Amplex Red catalase assay (EnzChek Myeloperoxidase Assay Kit with Amplex Ultra Red reagent; Invitrogen) and expressed as U/mg of protein.

### Calculation of Antioxidant Score

A global antioxidant score was calculated for each sample taking into account the abovementioned antioxidant parameters (GSH, total thiols, SOSA, and catalase activity) as previously described (Ruiz-Hurtado et al., 2014). Normality analysis for the different biomarkers was analyzed through the Kolmogorov–Smirnov test. The parameters that did not show a normal distribution were normalized through logarithmic transformation and standardized according to the following formula:

$$Z_{\text{ij}}=(X_{\text{ij}}-M_{\text{j}})/S_{\text{j}}$$

where: *Z*_ij_ is the standardized value of the specific biomarker j of sample i; *X*_ij_ is the raw measure (normal or log-transformed) of each biomarker j of samplei; *M*_j_ is the mean value of each biomarker j; and *S*_j_ is the Standard Deviation of each biomarker j.

### Statistical Analysis

Contractile responses are expressed as the percentage of contraction produced by 75 mmol/L KCl. Relaxations are expressed as the percentage of contraction produced by NA (10^-7^–10^-6^ M). The maximal response (*E*_max_ values) and the potency (negative logarithm of concentration) of Ach, NA, or Ang II producing 50% of maximum response (pD2 values) were determined by a non-linear regression analysis of each individual concentration-response curve. Area under the concentration-response curves (AUC) were calculated from the individual concentration response curve plots (GraphPad Software, California, United States). All values are given as mean ± SEM. One-way ANOVA followed by Newman–Keuls *post hoc* tests were used. A value of *p* < 0.05 was considered statistically significant. Statistical analyses were performed with Stat View software (SAS Institute, United States).

## Results

**FIGURE 1:**
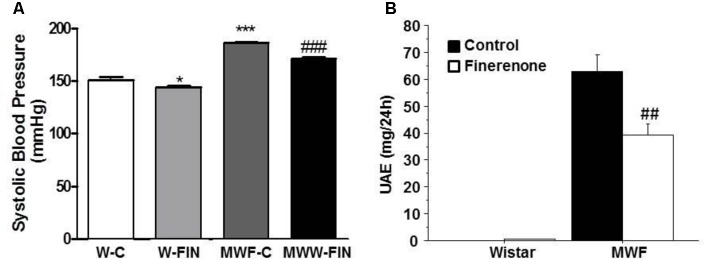
Effect of chronic treatment with finerenone on systolic blood pressure (SBP) and albuminuria. Systolic blood pressure **(A)** and urinary albumin excretion (UAE, **B**) in control and finerenone-treated rats. Data are represented as mean ± SEM for 10 animals per group. ^∗^*p* < 0.05; ^∗∗∗^*p* < 0.001 compared to W-C. ^##^*p* < 0.01; ^###^*p* < 0.001 compared to MWF-C. W, Wistar Kyoto; MWF, Munich Wistar Frömter; C, control; FIN, finerenone.

SBP and MAP were significantly higher in MWF at the end of the study compared to aged-matched W (**Figure 1A** and **Table 1**). Treatment with finerenone significantly reduced SBP in MWF rats, although SBP was still higher in MWF-FIN compared to W-C.

**Table 1:** Body weight, kidney weight, and mean arterial blood pressure values in experimental groups.

	W-C	W-FIN	MWF-C	MWF-FIN
Body weight (g)	391,8 ± 17,2	392,5 ± 12,7	362,8 ± 12,8	350,3 ± 9,0
Kidney weight (g/cm tibia)	1,1 ± 0,1	1,1 ± 0,04	1,1 ± 0,04	1,0 ± 0,03
Mean arterial blood pressure (mmHg)	87.2 ± 3.2	88.3 ± 4.3	128.9 ± 3.6^∗∗^	118.1 ± 4.7^#^

*Data are expressed as mean ± SEM, n = 10. ^∗∗^p < 0.01, compared to the W-C group. ^#^p < 0.05 compared with MWF-C. W, Wistar Kyoto; MWF, Munich Wistar Frömter; C, control.*

Finerenone lead to a significant reduction (>40%) in albuminuria in the MWF model (**Figure 1B**), while normoalbuminuria was not affected in Wistar rats. No effect of treatment was observed on body weight or kidney weight (**Table 1**).

Median unbound finerenone plasma concentrations were found to be 24.2 μg/L in MWF-FIN animals and 34.09 μg/L in W-FIN animals confirming that finerenone plasma levels were high enough to cover finerenone’s in vitro IC_50_ (18 nM) at MR. Plasma levels in Wistar-C and MWF-C groups at baseline were similar (1247 ± 349 and 1193 ± 213 pM).

### Finerenone Significantly Improves Endothelial Function in MWF Rats Through an Increase in Endothelial Nitric Oxide Availability

**FIGURE 2:**
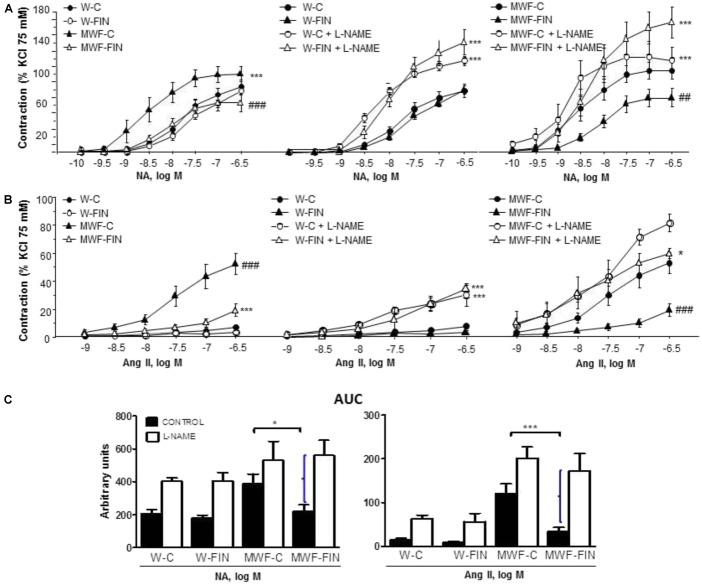
Effect of chronic treatment with finerenone on basal nitric oxide (NO) release. Concentration-response curves to **(A)** noradrenaline (NA, 10^-10^–5 × 10^-7^ mol/L) or **(B)** angiotensin II (Ang II, 10^-9^–5 × 10^-7^ mol/L) in aortic rings from W and MWF rats in absence an presence of L-NAME (10^-4^ mol/L). Data are shown as mean ± SEM of 10 animals per strain. **(C)** AUC, Area under concentration–response curves elicited by NA or Ang II in presence/absence L-NAME. AUC is expressed in arbitrary units. Curley bracket indicates NO availability. ^∗^*p* < 0.05; ^∗∗∗^*p* < 0.001 compared to their corresponding matched control groups. ^###^*p* < 0.001; ^##^*p* < 0.01 compared to their corresponding matched finerenone group. W, Wistar Kyoto; MWF, Munich Wistar Frömter; C, control; FIN, finerenone.

The contractile effect of 75 mM KCl, which indicates contractile capacity of the aorta, was similar between groups (W-C = 1.72 ± 0.1 g; W-FIN = 1.94 ± 0.1 g; MWF-C = 1.82 ± 0.1 g; MWF-FIN = 1.95 ± 0.1 g; n.s.). The overall contractile effect of NA (10^-10^–5 × 10^-7^ mol/L; **Figure 2A**) or Ang II (10^-9^–5 × 10^-7^ mol/L; **Figure 2B**) was significantly higher in arteries from MWF affecting both *E*_max_ and pD2 values (**Table 2**) as compared to W-C (*p* < 0.001). Treatment with finerenone significantly decreased contractile response to NA (**Figure 2A** and **Table 2**) or Ang II (**Figure 2B** and **Table 2**) in MWF-FIN to control levels. No effect of finerenone was observed in the W strain.

**Table 2:** E_max_ and pD_2_ values of Ach-induced relaxation and NA or Ang II-induced contractions in aorta.

		W-C	W-FIN	MWF-C	MWF-FIN
Ach	E_max_	82.0 @ 1.3	85.1 @ 1.3	75.8 @ 1.8**ˆ###	83.3 @ 1.5ˆ&&&
	pD_2_	7.0 @ 0.1	7.2 @ 0.1	6.5 @ 0.1***	6.7 @ 0.1*ˆ### &
NA	E_max_	80.3 @ 5.2	79.5 @ 8.8	98.1 @ 10.3	64.5 @ 11.6ˆ&&&
	pD_2_	7.6 @ 0.1	7.4 @ 0.1	8.1 @ 0.1***	7.8 @ 0.1*ˆ##&
Ang II	Emax	15.5 @ 6.8	5.1 @ 5.7	51.9 @ 7.3***ˆ###	19.0 @ 4.4ˆ&&&
	pD_2_	7.2 @ 0.2	7.8 @ 0.2	7.3 @ 0.2	7.1 @ 0.2

*E_max_, is the maximal relaxation to Ach expressed as % of precontraction to noradrenaline (NA), as well as the maximal contraction to NA or angiotensin II (Ang II) expressed as % of contraction to 75 mM KCl. pD_2_, is the negative logarithm of molar concentration of Ach, NA, or Ang II causing half maximal responses. Data are expressed as mean ± SEM, n = 10. ^∗∗^p < 0.01, ^∗∗∗^p < 0.001, compared to the W-C group. ^##^p < 0.01,^###^p < 0.001 compared to the W-FIN group. ^&^p < 0.05, ^&&&^p < 0.001, compared to the MWF-C group. W, Wistar Kyoto;Munich Wistar Frömter; C, control; FIN, finerenone; Ach, acetylcholine*.

Incubation with L-NAME to assess basal NO availability significantly increased contraction to NA (**Figure 2A**) or Ang II (**Figure 2B**) in both W-C and W-FIN without differences between groups. However, L-NAME significantly increased contractions to NA (**Figure 2A**) or Ang II (**Figure 2B**) in aortic rings from finerenone-treated MWF but not in untreated MWF. The difference in area under the concentration-response curves (AUC; **Figure 2C**) in absence and presence of L-NAME, which indirectly indicates basal NO availability, was significantly higher in finerenone-treated MWF for both NA and Ang II compared with untreated MWF (**Figures 2C**).

**FIGURE 3:**
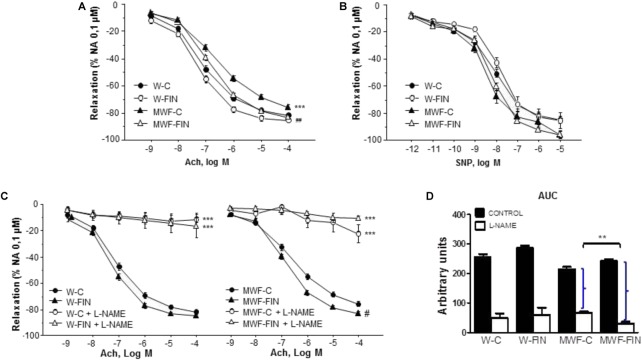
Effect of chronic treatment with finerenone on endothelial-dependent relaxation. Concentration-response curves to **(A)** acetylcholine (Ach, 10^-9^–10^-4^ mol/L) or **(B)** sodium nitroprusside (SNP, 10^-12^–10^-5^ mol/L) in aortic rings from W and MWF rats, **(C)** Effect of L-NAME (10^-4^ mol/L) on Ach-induced relaxation aortic rings from W rats and MWF rats. **(D)** AUC, Area under concentration–response curves elicited by Ach in presence/absence L-NAME. AUC is expressed in arbitrary units. ^∗∗^*p* < 0.01; ^∗∗∗^*p* < 0.001 compared to their corresponding matched control groups. ^##^*p* < 0.01; ^#^*p* < 0.05 compared to their corresponding matched finerenone group. Data are shown as mean ± SEM of 10 animals per strain. L-NAME, NG-nitro-L-arginine methyl ester; W, Wistar Kyoto; MWF, Munich Wistar Frömter; C, control; FIN, finerenone.

Next, we analyzed the effect of finerenone on endothelial-dependent relaxation (**Figure 3**). Ach (10^-9^–10^-4^ mol/L) induced a concentration-dependent relaxation in aorta from MWF-C that was significantly shifted to the right compared to W-C rings, with a significant decrease in both maximal response (*E*_max_) and potency (pD2) compared with W-C (**Figure 3A** and **Table 2**). Finerenone treatment significantly increased relaxation to Ach (*E*_max_ and pD2) in both MWF-FIN and W-FIN (**Figure 3A** and **Table 2**). No differences between strains or treatment were observed in endothelium-independent relaxation elicited by sodium nitroprusside (10^-12^–10^-5^ mol/L, **Figure 3B**). L-NAME almost abolished relaxation to Ach in all groups (**Figure 3C**). However, the difference between AUC (**Figure 3C**) in absence and presence of L-NAME was significantly higher in MWF-FIN compared to MWF. No significant effect was observed in W-FIN compared to W-C.

### Finerenone Reduces Superoxide Anion and Hydrogen Peroxide Availability in the Vascular Wall

**FIGURE 4:**
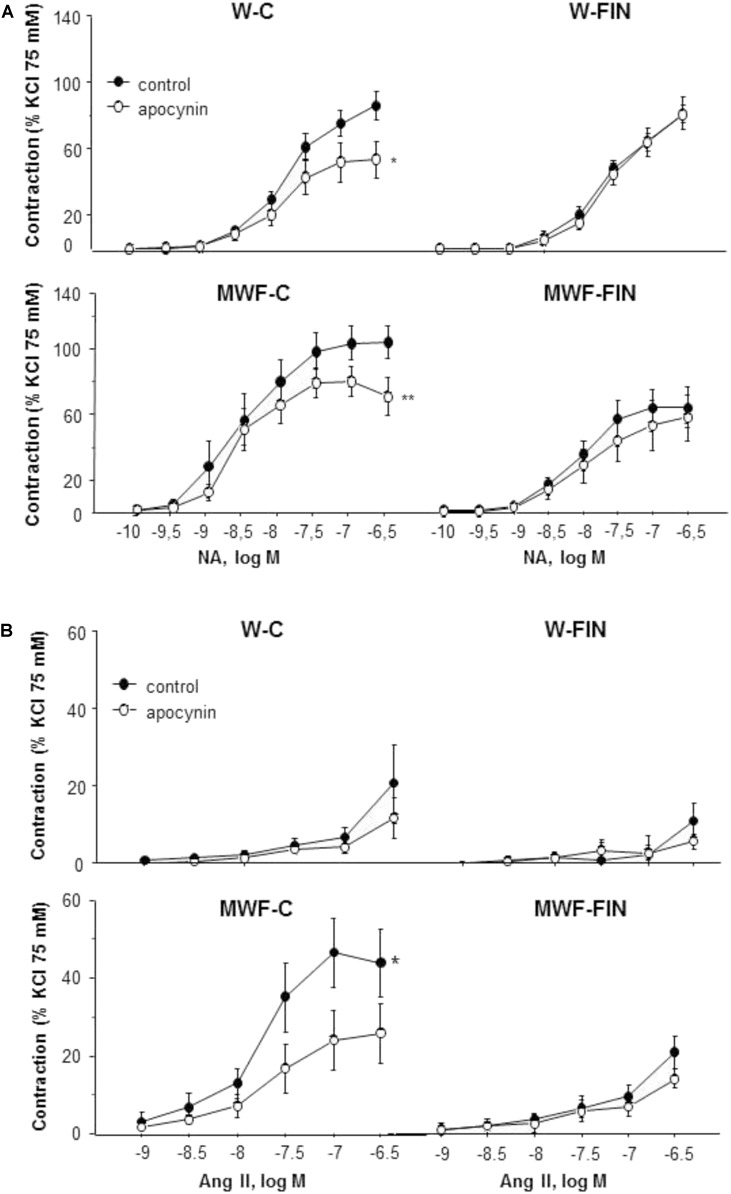
Contribution of superoxide anions on contractile responses to noradrenaline and angiotensin II. Effect of apocynin (10^-4^ mol/L) on cumulative concentration-response curves to **(A)** NA and **(B)** Ang II in aortic rings from W and MWF rats. Data are shown as mean ± SEM of eight animals per strain. ^∗∗^*p* < 0.01; ^∗^*p* < 0.05 compared to their corresponding matched control groups. W, Wistar Kyoto; MWF, Munich Wistar Frömter; C, control; FIN, finerenone.

To analyze the effect of finerenone treatment on superoxide anion availability, rings were preincubated with apocynin (10^-4^ mol/L), an inhibitor of NADPH oxidase. Apocynin significantly reduced NA-induced contractions in both untreated W and MWF rats (**Figures 4A,B**). However, apocynin had no significant effect on contractions to NA in finerenone-treated W and MWF (**Figure 4A**). Similar results were observed for Ang II-induced contractions (**Figure 4B**), were apocynin reduced contractions only in MWF rings.

**FIGURE 5:**
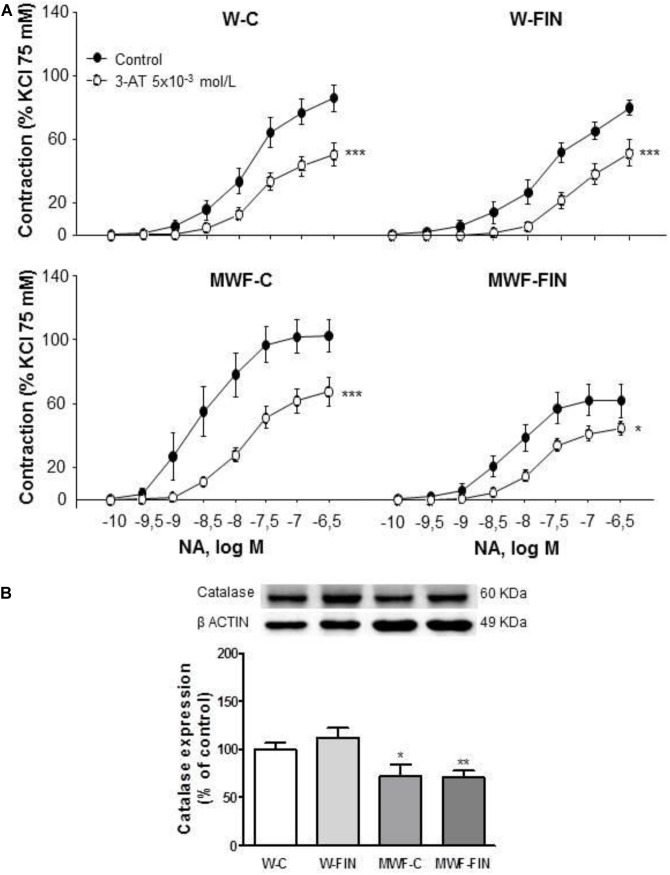
Contribution of hydrogen peroxide on contractile response to noradrenaline. **(A)** Effect of 3-aminotriazole (3-AT 5 × 10^-3^ mol/L) on cumulative concentration-response curves to NA in aortic rings from W and MWF rats. **(B)** Representative inmunoblot of catalase and densitometric analysis expressed as percentage of catalase/β-actin in the control group. Data are shown as mean ± SEM of eight animals per strain. ^∗∗∗^*p* < 0.001; ^∗^*p* < 0.05 compared to their corresponding matched control groups. ^#^*p* < 0.05 compared to their corresponding matched finerenone group. W, Wistar Kyoto; MWF, Munich Wistar Frömter; C, control; FIN, finerenone.

The functional effects of endogenous hydrogen peroxide were assessed after preincubation with the catalase inhibitor, 3-aminotriazole (3-AT, 5 × 10^-3^ mol/L). 3-AT significantly reduced NA-induced contractions in all groups (**Figure 5A**). However, the difference in AUC for NA in presence and absence of 3-AT was significantly higher in MWF-C (MWF-C; AUC_C_= 203.3 ± 31.3 vs. AUC_3-AT_= 93.2 ± 12.7, *p* < 0.01; W-C; AUC_C_= 100.2 ± 12.8 vs. AUC_3-AT_= 59.9 ± 9.9, *p* < 0.05) and reduced to control levels after FIN treatment (MWF-F; AUC_C_= 104.8 ± 20.0 vs. AUC_3-AT_= 61.6 ± 7.9, *p* > 0.05). Catalase levels were significantly lower in MWF compared to W and were not modified by finerenone treatment in either strain (**Figure 5B**).

### Finerenone Treatment Induces an Upregulation of Mn-SOD, Cu/Zn-SOD, and Total SOD Activity, as Well as pAkt and peNOS in the Vascular Wall

**FIGURE 6:**
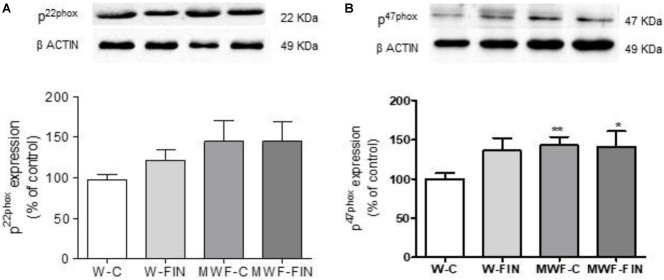
**(A)** Representative inmunoblot of p22 phox and densitometric analysis expressed as percentage of p22phox/β-actin in the control group. **(B)** Representative inmunoblot of p47 phox and densitometric analysis expressed as percentage of p47phox/β-actin in the control group. Data are shown as mean ± SEM of 10 animals per strain. ^∗^*p* < 0.05; ^∗∗^*p* < 0.01 compared to W-C. W, Wistar Kyoto; MWF, Munich Wistar Frömter; C, control; FIN, finerenone.

To investigate whether reduced superoxide anion availability is due to a downregulation of NADPH oxidase levels we measured protein levels of NADPH oxidase subunits, p22-phox and p47-phox. No differences were observed between groups for p22phox (**Figure 6**). In contrast, p47phox was significantly higher in MWF compared to W, but was no modified by treatment (**Figure 6**).

**FIGURE 7:**
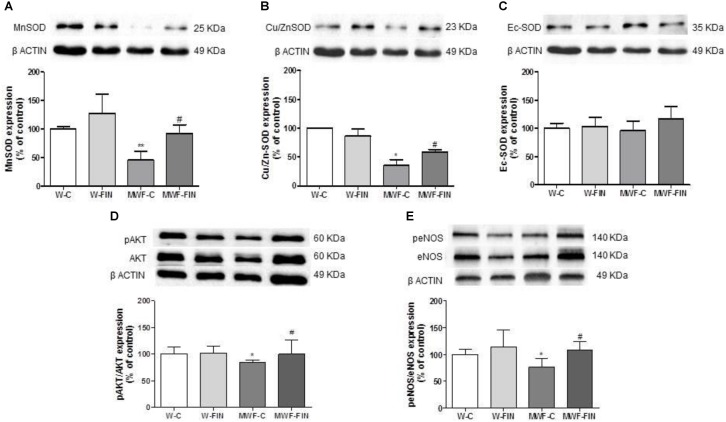
Effect of finerenone on superoxide dismutase (SOD) isoforms, pAkt and peNOS levels. Representative inmunoblot and densitometric analysis of: **(A)** MnSOD and **(B)** CuZnSOD, and **(C)** EcSOD expressed as percentage of β-actin in the control group. The β-actin loading control has been reused in **A** and **B**. **(D)** Representative inmunoblot of pAkt (Ser^473^) and densitometric analysis expressed as percentage of pAkt (Ser^473^)/Akt in the control group. **(F)** Representative inmunoblot of peNOS (Ser^1177^) and densitometric analysis expressed as percentage of peNOS (Ser^1177^)/eNOS in the control group. Data are mean ± SEM (*n* = 5). ^∗∗^*p* < 0.01; ^∗^*p* < 0.05 compared to their corresponding matched control groups. ^#^*p* < 0.05 compared to their corresponding matched finerenone group. W, Wistar Kyoto; MWF, Munich Wistar Frömter; C, control; FIN, finerenone.

We next analyzed if reduced hydrogen peroxide availability was due to changes in SOD. Both Mn-SOD (**Figure 7A**) and Cu/Zn-SOD (**Figure 7B**) levels were significantly lower in the untreated MWF and upregulated by finerenone treatment, although they did not reach W-C levels. No differences were observed in ec-SOD levels between groups (**Figure 7C**).

Levels of pAkt/Akt and peNOS^Ser1177^/eNOS (**Figures 7D,E**) were smaller in control MWF and significantly upregulated by finerenone treatment.

### Pro-oxidant and Anti-oxidant Markers in Kidney and Plasma

Renal total SOD activity was lower in MWF-C (51.9 ± 3.4 U/mg prot, *p* < 0.05) compared to W-C (63.7 ± 2.4 U/mg prot) rats, but was significantly increased in MWF-FIN (60.9 ± 4.0 U/mg prot, *p* < 0.05) up to control levels. Catalase activity, thiol groups, and reduced glutathione (**Table 3**), measured as important non-enzymatic low-molecular-weight antioxidant systems, were similar between strains and not modified by treatment. Carbonyl and malondialdehyde (MDA) levels to estimate the oxidative damage index related to albuminuria were not different between strains nor modified by treatment (**Table 3**). Antioxidant score was significantly lower in MWF-C. There was no significant difference between MWF-FIN and W-C group, although no statistical difference could be observed in comparison with MWF-C either.

**Table 3:** Anti-oxidant and pro-oxidant markers in kidney.

Kidney	W-C	W-FIN	MWF-C	MWF-FIN
SOD activity (U/mg prot)	63.7 @ 2.4	54.1 @ 7.7	51.9 @ 3.4*	60.9 @ 4.0ˆ#
Catalase activity (U/mg prot)	2.4 @ 0.2	2.3 @ 0.2	2.4 @ 0.2	2.2 @ 0.2
Thiols (μM/mg prot)	21.1 @ 2.0	19.7 @ 2.8	17.4 @ 2.2	17.8 @ 1.4
GSH (mg/mg prot)	0.06 @ 0.01	0.1 @ 0.03	0.05 @ 0.01	0.05 @ 0.01
Carbonyls (nmol/mg prot)	1.2 @ 0.3	1.2 @ 0.2	1.0 @ 0.1	0.9 @ 0.1
Lipid peroxidation (μM)	8.9 @ 0.7	7.7 @ 0.9	5.8 @ 0.7	5.4 @ 1.1
Antioxidant score	0.32 @ 0.1	0.29 @ 0.2	0.01 @ 0.1*	0.11 @ 0.1

*Data are expressed as mean ± SEM, n = 10. ^∗^p < 0.05 compared to the W-C group. ^#^p < 0.05 compared to the MWF-C group. W, Wistar Kyoto; Munich Wistar Frömter; C, control; FIN, finerenone; SOD, superoxide dismutase; GSH, reduced glutathione*.

In plasma, total SOD activity was lower in MWF-C but not modified by treatment (**Table 4**). No statistical differences were observed in other anti- or pro-oxidant variables.

**Table 4:** Anti-oxidant and pro-oxidant markers in plasma.

Plasma	W-C	W-F	MWF-C	MWF-F
SOD activity (U/mg prot)	1.1 @ 0.2	0.7 @ 0.1	0.5 @ 0.05**	0.5 @ 0.05**
Catalase activity (U/mg prot)	2.2 @ 0.7	1.9 @ 2.5	2.9 @ 0.7	2.6 @ 0.8
Thiols (μM/mg prot)	2.8 @ 0.3	3.3 @ 0.3	3.2 @ 0.8	2.8 @ 0.8
GSH (mg/mg prot)	0.2 @ 0.04	0.3 @ 0.04	0.2 @ 0.03	0.2 @ 0.01
Carbonyls (nmol/mg prot)	0.83 @ 0.1	0.89 @ 0.2	1.24 @ 0.3	1.1 @ 0.2
Lipid peroxidation (μM)	8.0 @ 2.3	14.0 @ 3.7	30.0 @ 14.3	16.1 @ 5.5
Antioxidant Score	-0.33 @ 0.1	-0.36 @ 0.1	-0.49 @ 0.1	-0.15 @ 0.1ˆ#

*Data are expressed as mean ± SEM, n = 10. ^∗∗^p < 0.05 compared to the W-C group. ^#^p < 0.05 compared to the MWF-C group. W, Wistar Kyoto; Munich Wistar Frömter; C, control; SOD, superoxide dismutase; GSH, reduced glutathione*.

## Discussion

**FIGURE 8:**
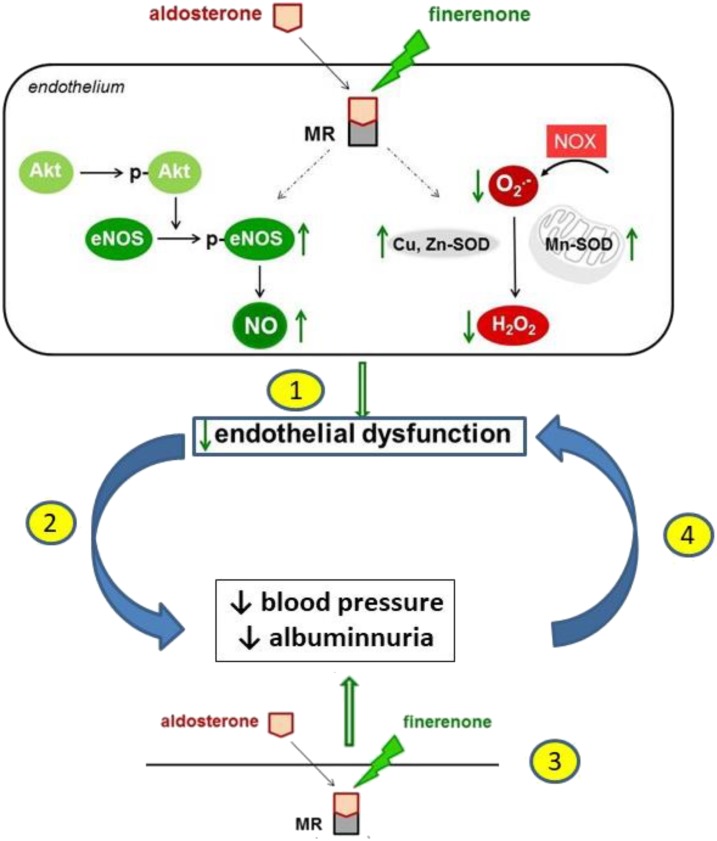
A schematic illustration of the proposed mechanism of action of finerenone at endothelial level leading to the improvement of endothelial dysfunction, lowering of blood pressure, and reduction of albuminuria. The sequence of events is not fully elucidated. The reduction of endothelial dysfunction by finerenone (1) might lead to a reduction of blood pressure and albuminuria (2). Alternatively, finerenone might lower blood pressure and albuminuria (3) leading to a reduction in endothelial dysfunction (4).

The present study demonstrates the efficacy of finerenone to ameliorate albuminuria and normalize endothelial dysfunction in the aorta of MWF rats at a blood pressure lowering dosage. These effects are related to an increase in endothelial NO availability due to an upregulation in peNOS^Ser1177^, Mn-SOD and Cu,Zn-SOD expression in the vascular wall with a subsequent decrease in superoxide anion and hydrogen peroxide levels. In addition, the upregulation of renal total SOD activity after finerenone treatment supports a functional link between extrarenal normalization of vascular dysfunction and improvement of glomerular permeability dysfunction (**Figure 8**).

Endothelial dysfunction in patients with albuminuria (Vaziri et al., 2002; Satoh, 2012; Ruiz-Hurtado et al., 2014) associates with increased oxidative stress in the vascular wall. Earlier studies in MWF rats confirmed an impaired endothelium-dependent relaxation (Gschwend et al., 2002b; Ulu et al., 2009; Szymanski et al., 2012; Steireif et al., 2013; Gil-Ortega et al., 2015) accompanied by widespread loss of the endothelial surface layer in mesenteric and glomerular microvessels (Hermans et al., 2008). Interestingly, impaired endothelial function is linked to high superoxide anion availability both in the vascular endothelium of arteries and within the kidney tissue (Steireif et al., 2013; Gil-Ortega et al., 2015). Thus, endothelial dysfunction associated to oxidative stress may represent a pathophysiological link between cardiovascular and renal injury. Of interest, genetic introgression of chromosome 8 from the spontaneously hypertensive rat (SHR), as a distinctive hypertension model with low-grade albuminuria, into the MWF background (MWF-8^SHR^) completely restored endothelial function, reduced abnormal O_2_^-^ production and significantly suppressed albuminuria in aorta (Steireif et al., 2013) and in mesenteric arteries (Gil-Ortega et al., 2015). This supports both a genetic and mechanistic link between albuminuria, endothelial dysfunction and oxidative stress.

The beneficial effect of finerenone on the restoration of endothelial function in MWF by an increase in peNOS^Ser1177^/eNOS, and thus in both basal and stimulated NO availability, is in accordance with previous data demonstrating that MR signaling contributes to endothelial dysfunction (Quaschning et al., 2001; Virdis et al., 2002; Sanz-Rosa et al., 2005; Bolignano et al., 2014). Moreover, finerenone has demonstrated beneficial effects on the endothelium, reducing endothelial cell apoptosis and re-endothelialization in a model of neointima formation (Dutzmann et al., 2017). In this study, finerenone also lowers superoxide anion levels by the reduction of NADPH oxidase activity, as functionally confirmed after apocynin preincubation, despite the lack of changes in the expression levels of p22phox and p47phox by treatment. Normalization of endothelial function by spironolactone (Quaschning et al., 2001) or eplerenone (Quaschning et al., 2001; Sanz-Rosa et al., 2005) through an increase in eNOS protein and the reduction of elevated NADPH oxidase activity was previously observed in several models of hypertension (Harvey et al., 2017). Dismutation of superoxide anion by the various SOD (Faraci and Didion, 2004) is a key step that leads to H_2_O_2_ formation, which is then metabolized by catalase to water. Both endothelial and smooth muscle cells produce H_2_O_2_ (Droge, 2002; Paravicini and Sobey, 2003). Interestingly, finerenone restores SOD activity in MWF due to an upregulation of cytosolic and mitochondrial SOD expression and reduces hydrogen peroxide levels as indicated by the observed response to 3-AT in our study. Spironolactone has been shown to reduce urinary H_2_O_2_ levels in renal transplant recipients (Ojeda-Cervantes et al., 2013).

The improvement of endothelial function and reduction of oxidative stress elicited by finerenone in MWF is associated with a significant reduction of albuminuria in MWF rats. The increased oxidative stress previously documented in kidney tissue of MWF (Steireif et al., 2013) supports the suggestion that vascular oxidative stress might be an indicator for similar dysfunctional changes at the glomerular level. Thus, albuminuria reduction by finerenone might be associated with the reduction of oxidative stress in the glomerulus, given that sufficient concentrations reach the kidney to achieve MR antagonism at the selected dosage used (Amazit et al., 2015). These beneficial effects of FIN in the MWF strain occurred despite the fact that aldosterone plasma levels in this strain are normal, i.e., similar to the Wistar strain. Similarly, in a rat model of acute ischemic kidney injury leading to CKD finerenone prevented reactive oxygen species generation during reperfusion and thus proteinuria, renal structural alterations and kidney damage (Lattenist et al., 2017).

Previous studies with spironolactone and eplerenone indicate that these compounds can improve microvascular endothelial function and reduce proteinuria in CKD (Zwadlo and Bauersachs, 2013; Bauersachs et al., 2015). However, the broad clinical use of steroidal MRAs is limited by the potential risk of inducing hyperkalemia when given on top of RAS blockade in patients with hypertensive and/or diabetic kidney disease (Briet and Schiffrin, 2013; Mavrakanas et al., 2014). Therefore, there is a need for novel, next generation non-steroidal MRAs. In the ARTS-DN trial, addition of finerenone to standard of care (i.e., ACEIs/ARBs) resulted in significant dose-dependent reductions in albuminuria at doses of 7.5, 10, 15, and 20 mg after 90 days of treatment in 823 randomized patients with T2DM and diabetic kidney disease (Bakris et al., 2015). Moreover, data from five clinical phase II trials with finerenone in more than 2000 patients with HF and additional CKD and/or diabetes as well as in patients with DKD have demonstrated that neither hyperkalemia nor reductions in kidney function were limiting factors to its use.

### Perspectives

Persistent albuminuria under chronic RAS blockade in well-controlled hypertensive patients is associated with an increase in plasma biomarkers for oxidative stress, which is not sufficiently counterbalanced by the endogenous antioxidant defense (Ruiz-Hurtado et al., 2014). In fact, higher albuminuria correlated with higher carbonyls as markers of oxidative protein damage (Ruiz-Hurtado et al., 2014). In a recent study among patients with diabetic nephropathy receiving an ACEi or an ARB, the addition of finerenone compared with placebo resulted in improvement in the urinary albumin-creatinine ratio (Bakris et al., 2015). Further clinical studies may evaluate whether a reduction in albuminuria and potentially oxidative stress can improve the outcome of hypertensive patients with albuminuria under chronic RAS suppression. In this regard, it appears of interest to await the results of two large phase 3 clinical outcome trials^1^ (NCT02545049 and NCT02540993) in patients with diabetic kidney disease in which the efficacy and safety of finerenone will be assessed.

## Author Contributions

RG-B and EV-M contributed to vascular function studies and analysis of the data. BS contributed to vascular function studies, blood pressure measurement, analysis of the data, and figures. MG-O contributed to western blot studies, study design, analysis of the data, and figures. MM-R contributed animal treatment and monitoring. DR-C contributed oxidative stress determinations and analysis of the data. AS contributed in the determination of albuminuria and analysis of the data. LR and PK designed the study, interpreted the critical results, and wrote the manuscript. RK and MF-A designed the study, supervised the experiments, interpreted the critical results, and wrote the manuscript.

## Conflict of Interest Statement

LR was advisor/speaker for Bayer Pharma AG, RK was supported by Bayer Research Grant and honorarium for consulting, MF-A was supported by Bayer Research Grant, and PK was an employee of Bayer AG. The remaining authors declare that the research was conducted in the absence of any commercial or financial relationships that could be construed as a potential conflict of interest. The handling Editor declared a shared affiliation, though no other collaboration, with the authors DR-C, LR at time of review.

## Footnotes

1 https://clinicaltrials.gov/
